# Resident Performance on the Obstetrics and Gynecology In-Training Examination After Implementation of a New Academic Curriculum

**DOI:** 10.7759/cureus.72861

**Published:** 2024-11-01

**Authors:** Kathleen R Lundeberg, Shannon Madison, Nancy Lo, Rose Maxwell, Jason Massengill

**Affiliations:** 1 Obstetrics and Gynecology, Wright State University Boonshoft School of Medicine, Dayton, USA

**Keywords:** creog, general obgyn, graduate medical education (gme), medical education & training, obgyn residents

## Abstract

Background

The goals of the annual Council on Resident Education in Obstetrics and Gynecology In-Training Examination (CREOG-ITE) are to provide residents with an assessment of their knowledge and program directors an assessment of their residency programs. Research has shown that a score greater than 200 is correlated with passing the qualifying board examination. We observed a substantial number of our residents were not performing well on the exam, which prompted the implementation of a new academic program aimed at determining the impact of an academic curriculum addition on CREOG-ITE scores in an American College of Graduate Medical Education (ACGME) accredited obstetrics and gynecology (OB/GYN) residency program.

Methods

We performed a prospective cohort study of residents at a single institution to evaluate the effect of a remediation course for residents identified as "at risk." Residents with CREOG-ITE scores of <180 were placed into the experimental group who underwent a year-long remediation program and were compared to the control group (those with scores >180 who did not undergo remediation). Each resident in the experimental group served as their internal control by comparing their pre-intervention score to their post-remediation score.

Results

The mean improvement of the experimental group of the remediation program was 23.2 points. After controlling for post-graduate year (PGY), their score improvement remained significant and was not simply a by-product of the inherent academic knowledge of a resident further along in training.

Conclusions

This scholastic remediation program was associated with a statistically significant improvement in CREOG-ITE scores, even when accounting for PGY level. This program may serve as a model for improving OB/GYN resident education.

## Introduction

A residency program’s goal is to train competent board-certified physicians. In 1967, the Council on Residency Education in Obstetrics and Gynecology (CREOG) convened for the first time to address this goal. A subcommittee known as the Committee on In-Training Examinations for Residents in Obstetrics and Gynecology (CITROG), created a standardized in-training examination (ITE) [1]. This examination was first administered in 1970 as a voluntary examination consisting of 477 multiple-choice questions [2,3]. The purpose of the examination was to provide residents with an assessment of their knowledge, highlight areas for improvement, and provide program directors with an assessment of their residency programs [4,5].

The annual CREOG-ITE is taken during the third week of January by every resident in an Accreditation Council for Graduate Medical Education (ACGME)-accredited Obstetrics and Gynecology (OB/GYN) residency training program and is the current method used to assess competence over the four years of their training [6]. In addition, performance on the CREOG-ITE is used as a measure of the effectiveness of the residency training program. CREOG, in conjunction with the American College of Obstetrics and Gynecology (ACOG) and the American Board of Obstetrics and Gynecology (ABOG), has set forth specific educational objectives for residents to master before completing their post-graduate residency training. The questions on the CREOG-ITE assess residents’ mastery of these key educational objectives. While some research has shown that scores on the CREOG-ITE are well correlated with scores on the ABOG qualifying examination, other research has shown a weak positive correlation but a strong negative predictive value for scores greater than 187.5 [7,8]. Another study by Lingenfelter et al demonstrated a CREOG-ITE score greater than 200 points is correlated to passing the ABOG qualifying examination, which is requisite to board certification [9].

Although there are multiple requirements for ACGME-accredited OB/GYN residency programs, there are no specific mandates for how the didactic portion of residents’ education is conducted. Numerous programs utilize weekly lectures, while some incorporate weekly quizzes to enhance learning, though no official recommendations exist for how to appropriately remediate residents deemed "at risk" [10-12]. Here we present the impact of implementation of a remediation program on CREOG-ITE scores for residents with a score of 180 or less, or one standard deviation below the mean, at a single institution which can be an applied strategy for other residency programs.

## Materials and methods

**Figure 1:**
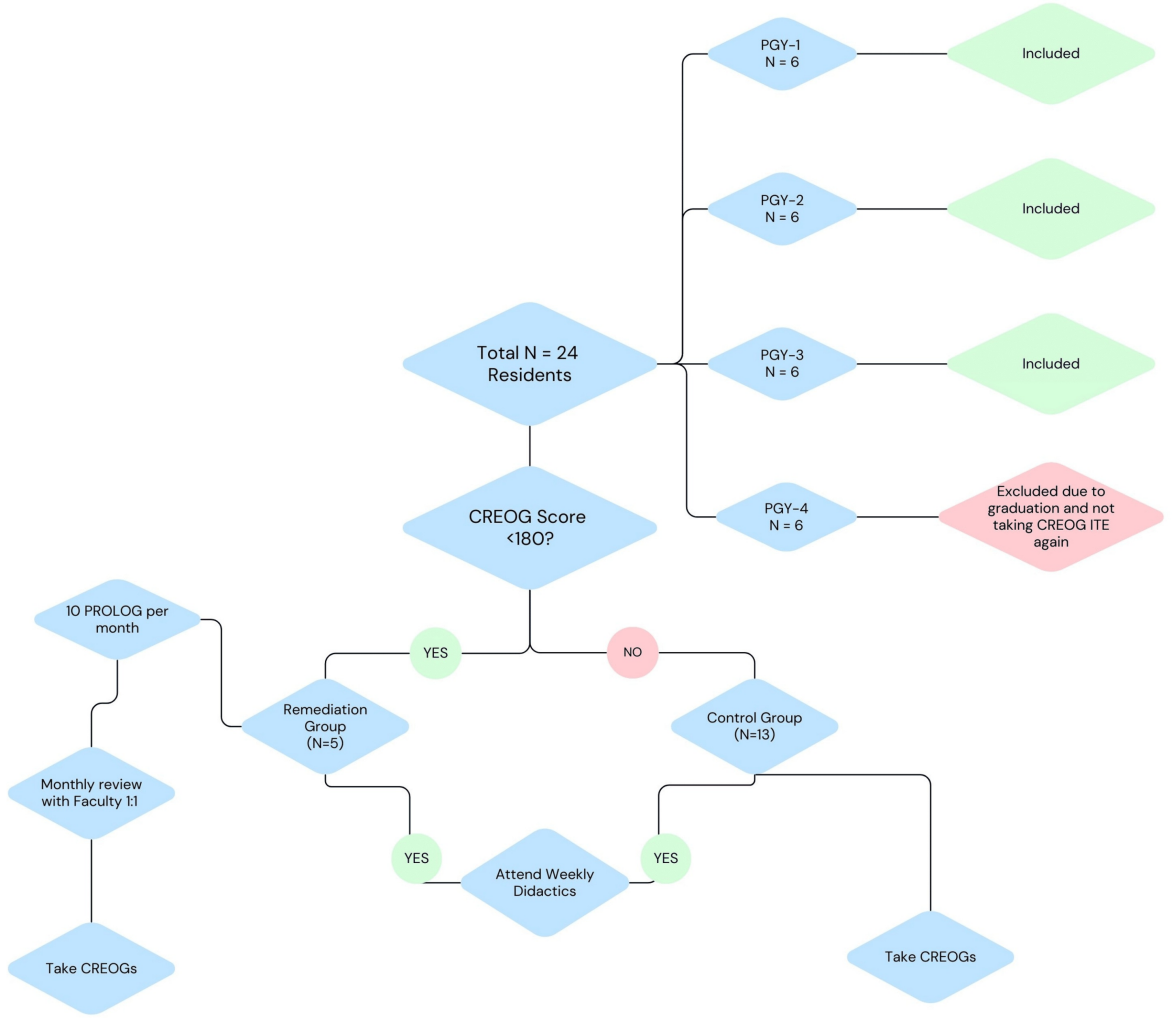
Remediation program selection and study design. OB/GYN residency programs are four years long, with varying resident cohorts varying per program. (PGY: postgraduate year). The remediation group of residents completed monthly sessions meeting one-on-one with a faculty mentor, completing and reviewing 10 PROLOG questions per month in addition to the weekly didactics session that is required of all residents. CREOG: Council on Residency Education in Obstetrics and Gynecology; ITE: In-training Examination; PROLOG: Personal Review of Learning in Obstetrics and Gynecology

This prospective cohort study analyzed the results of the pilot CREOG-ITE remediation program at a single institution to evaluate the effect of a remediation course for residents identified as "at risk." This study was approved by the Wright State University Institutional Review Board (IRB) and deemed exempt as a quality improvement study (#06024). The annual January CREOG-ITE scores for our 24 OB/GYN residents (six residents per PGY class in a four-year residency) were compared the year before and the year after the implementation of this remediation program. The residents with a score of one standard deviation below the mean or less than 180 points were identified and placed into the experimental group (n = 5) with resident permission. The residents with a score greater than 180 were designated as the control group (n = 13). Exclusion criteria included graduating residents who would not be taking the CREOG-ITE again, leaving a total of 18 residents meeting the criteria for inclusion (n = 18, Figure 1).

The remediation program for the experimental group involved monthly quizzes consisting of ten standardized Personal Review of Learning in Obstetrics and Gynecology (PROLOG) questions carefully selected from the six-volume learning resource on key topics in obstetrics and gynecology as outlined by the CREOG Educational Objectives. The experimental group was also required to meet with a faculty member every month to discuss study habits and the answers to the selected ten PROLOG questions. The control group received no additional enforced formal education outside of weekly scheduled didactics lectures which all residents attended.

Each resident in the intervention group served as their own matched paired internal control by comparing their first score to their score the following year after the remediation program. Paired analysis was performed using nonparametric due to the small study cohort of 18 residents (SPSS for Windows, version 16.0, Chicago, Ill; 2007). A power analysis of a paired t-test, assuming an alpha of 0.05 and power of 80%, indicated a sample size of six total subjects would be needed to detect a statistically significant difference before and after the institution of the remediation pilot program. Significance was set at p-value < 0.05. 

## Results

**Figure 2:**
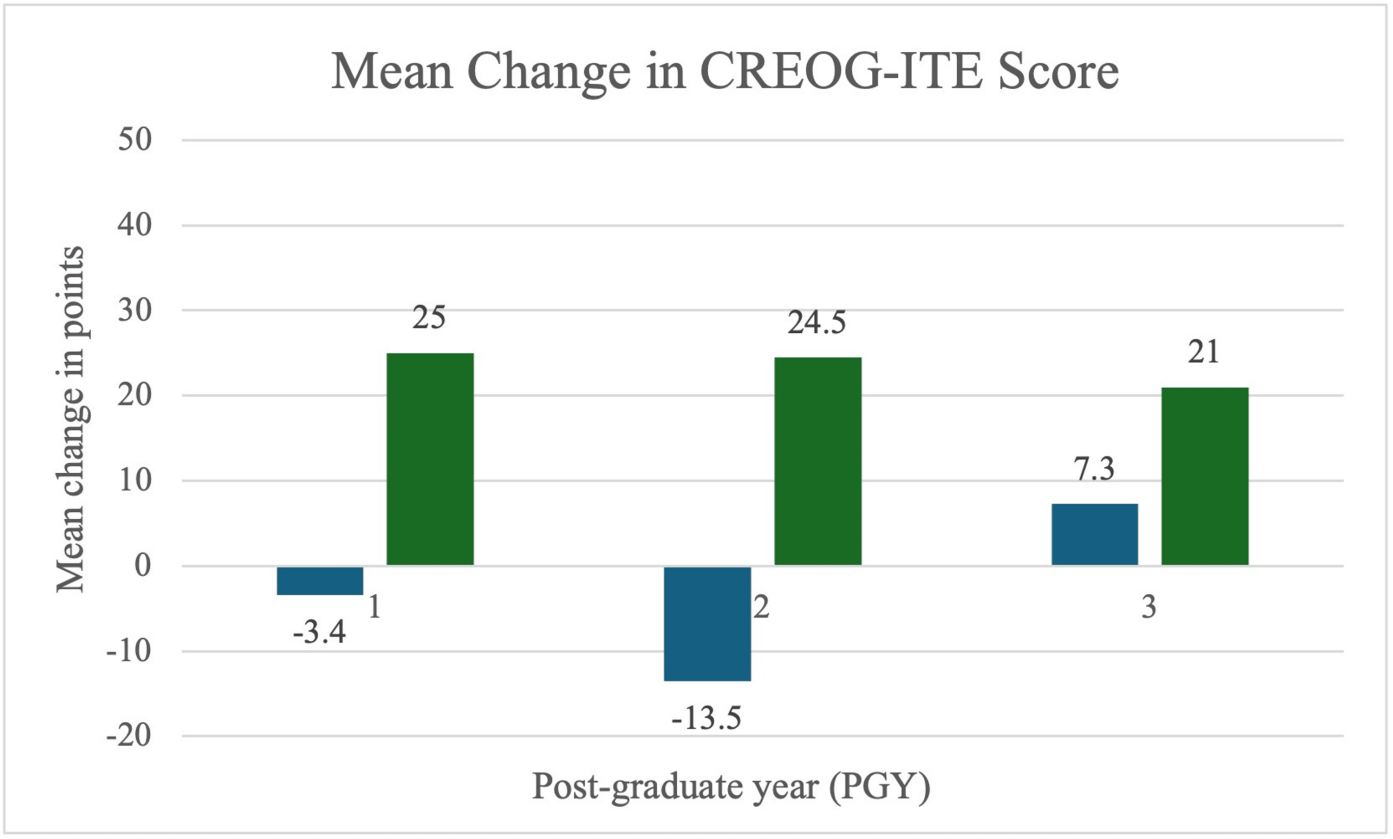
Change in CREOG-ITE scores by PGY level. Blue: control group; Green: experimental group. PGY: postgraduate year; CREOG: Council on Residency Education in Obstetrics and Gynecology; ITE: In-training examination

The mean score for our program on the pre-intervention CREOG-ITE was 191.5 points, with a standard deviation of +/- 19 points. Comparatively, the mean score post-intervention was 196.4 with a standard deviation of +/- 16 points. The demographics of residents included in each cohort are listed in Table 1, with no significant difference in age, race, or gender. The experimental group demonstrated a mean improvement of 23.2 points (Table 2), which was statistically significant compared to the control group’s decrease of 3.2 points (95% Confidence Interval 12.4 to 40.5; p = 0.001). Analysis by PGY level demonstrated an overall increase in points for those who completed the remediation program while those in the control group experienced a decrease in points (Table 3, Figure 2). When controlling for the groups by residential training year, the mean improvement remained statistically significant (Table 4).

**Table 1:** Demographics of Control and Remediation Groups Statistical significance was set at p-value < 0.05.

	Control	Remediation	p-value
Gender			0.56
Male	1	1	-
Female	12	4	-
Race			0.28
Caucasian	13	5	-
Other	0	0	-
Average Age	30.6	29.5	0.91

**Table 2:** Comparison of CREOG-ITE scores. SD: standard deviation; CREOG: Council on Residency Education in Obstetrics and Gynecology Statistical significance was set at p-value < 0.05 with * denoting significance.

	Number	Mean Change in Points (SD)	p-value	Confidence Interval
Control	13	-3.2 (12.9)	-	-
Pilot Program	5	23.2 (11.5)	-	-
Total	18	26.4	0.001*	12.4 to 40.5

**Table 3:** Change in CREOG-ITE scores stratified by training year. N: number; PGY: postgraduate year; SD: standard deviation; CREOG: Council on Residency Education in Obstetrics and Gynecology; ITE: In-training examination

Control	N = 13	Mean Change in Points	SD
PGY4	5	-3.4	9.3
PGY3	4	-13.5	13.8
PGY2	4	7.3	8.5
Pilot Program N = 5			
PGY4	1	25	0
PGY3	2	24.5	14.8
PGY2	2	21	16.9

**Table 4:** CREOG-ITE scores controlling for training level PGY: postgraduate year; CREOG: Council on Residency Education in Obstetrics and Gynecology; ITE: In-training examination Statistical significance was set at p-value < 0.05 with * denoting significance.

	p-value
Participation in the Pilot Program	0.001*
Effect of PGY Level on the Outcome of the Pilot Program	0.275

## Discussion

The CREOG-ITE was developed to provide an annual assessment for residents and training programs to develop competent, board-certified OB/GYNs. Our institution developed a remediation program to augment formal didactics training which significantly improved the CREOG-ITE scores in the experimental group who completed the pilot program. Research has shown that initiation of a structured academic curriculum ranging from weekly quizzes, resident-created study guides, or textbook chapter reviews coupled with presentations is correlated with improved CREOG-ITE scores in programs with scores below the national average, but this was not necessarily statistically significant among all PGY levels [13-15].

Although our sample size was small, the implications of a statistically significant difference in CREOG-ITE score for the residents in the scholastic remediation pilot program are notable. When accounting for PGY-level as a potential confounding variable, the improvement in CREOG-ITE scores for residents in the scholastic remediation pilot program remained significant. This improvement is likely not simply attributable to monthly quizzes, but also attributable to improved study habits and personal accountability associated with a one-to-one monthly meeting with a faculty member.

One finding of note is the lack of improvement in our control group. While further investigation into this is warranted, we postulate this could be attributed to a shift in focus toward honing surgical skills in the final two years of residency, which are indirectly assessed by the CREOG-ITE. Another possible explanation is that our program is structured such that most core OB/GYN rotations, except for Urogynecology and Reproductive Endocrinology, and Infertility, have been covered by the end of the PGY-2 year. We postulate this could potentially lead to a plateau in a fund of knowledge, attributing to the lack of improvement in CREOG scores after the PGY-2 level. Similar results have been noted in prior studies examining CREOG-ITE scores and have been attributed to a plateau in knowledge [4].

The strengths of our study include having internally matched controls for the experimental group and a large statistically significant improvement in CREOG-ITE scores. While our study included a small sample size with only five (n=5) in the experimental group and thirteen (n=13) in the control group, a power analysis indicated a sample size of six total subjects would be needed to detect a statistically significant difference. This scholastic remediation pilot program was limited to only one institution and may not be as easily translated to larger institutions with more than 6 residents per class. However, institutions with larger resident classes typically also have larger faculty panels which would inherently be equipped to support this remediation program. Additionally, as the CREOG-ITE scores at our program were below the national average, other programs with average or above average CREOG-ITE scores might not experience as significant of an improvement with the implementation of a similar program.

While only residents with a CREOG-ITE score <180 or one standard deviation below the mean were assigned to the scholastic remediation pilot program, it is possible that these residents could have been more intrinsically motivated to study than residents with scores greater than 180, accounting for the improvement. An area for further research could include examining change in CREOG-ITE scores over 1 year in residents with scores <180, before the implementation of the scholastic remediation pilot program, to eliminate intrinsic desire to improve as a possible explanation for the large increase in CREOG-ITE scores. Additional areas for future research include continuing to follow residents’ progress in CREOG-ITE performance after two years in the scholastic remediation pilot program, as well as opening the program to all residents, regardless of initial CREOG-ITE score.

## Conclusions

The success of our remediation pilot program for OB/GYN residents preparing for the CREOG-ITE is promising. We have continued this remediation program for residents with scores <180 or more than one standard deviation below the mean, and have expanded the study program to our PGY-1 class as a scholastic preparation program that institutes a culture of effective self-study at an early stage of residency training. We hope that our institution’s scholastic remediation pilot program can serve as a model for other ACGME-accredited OB/GYN residency programs desiring to improve residents’ CREOG-ITE scores, and ultimately improve the ABOG qualifying examination pass rate.
